# Crystal structure of 3,5-di­methyl­phenyl 2-nitro­benzene­sulfonate

**DOI:** 10.1107/S2056989015015078

**Published:** 2015-08-22

**Authors:** Tsvetelina P. Atanasova, Sean Riley, Shannon M. Biros, Richard J. Staples, Felix N. Ngassa

**Affiliations:** a Department of Chemistry, Grand Valley State University, 1 Campus Dr., Allendale, MI 49401, USA; b Center for Crystallographic Research, Department of Chemistry, Michigan State University, 578 S. Shaw Lane, East Lansing, MI 48824, USA

**Keywords:** crystal structure, nitro­benzene­sulfonate, N(nitro)⋯O inter­actions, C—H⋯O inter­actions, π–π inter­actions

## Abstract

The title compound, C_14_H_13_NO_5_S, was synthesized *via* a nucleophilic substitution reaction between 3,5-di­methyl­phenol and 2-nitro­benzene­sulfonyl chloride. The aromatic rings attached to the SO_3_ group are oriented in a *gauche* fashion around the ester S—O bond, with a C—S—O—C torsion angle of 84.68 (11)°. The mol­ecules form centrosymmetric dimers *via* π–π stacking inter­actions between 3,5-di­methyl­phenyl groups (centroid–centroid distance = 3.709 Å). An inter­molecular S=O⋯N inter­action between the sulfonyl and nitro groups, with an O⋯N distance of 2.9840 (18) Å, organizes the dimers into columns extending along [011]. These columns are further assembled into (111) layers through C—H⋯O inter­actions.

## Abstract 2

In the title compound, there are inter­molecular S=O⋯N(nitro) inter­actions, with an O⋯N distance of 2.9840 (18) Å, between inversion-related mol­ecules. The aromatic rings attached to the SO_3_ group are oriented in a *gauche* fashion around the ester S—O bond, with a C—S—O—C torsion angle of 84.68 (11)°.

## Chemical context   

Microtubules form a major component of the cytoskeleton and have been implicated in a wide variety of cellular functions, such as cell division (Jordan & Wilson, 2004 ▸). Microtubules therefore have been targeted in the design of drugs for the treatment of various forms of cancer (Spencer & Faulds, 1994 ▸; Teicher, 2008 ▸; Trivedi *et al.*, 2008 ▸). For example, Combretastatin A-4 (CA-4) has been shown to target tumor vasculature (Griggs *et al.*, 2001 ▸). Most known anti­microtubules have poor biopharmaceutical properties, uch as chemoresistance and toxicity (Islam *et al.*, 2003 ▸; Fortin *et al.*, 2011 ▸).
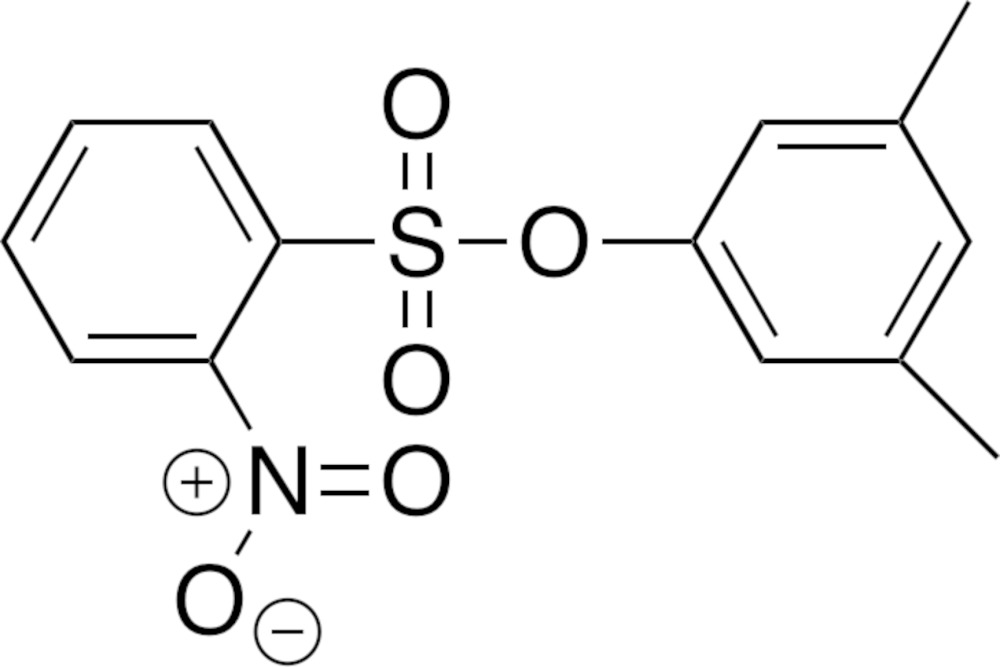


**Figure 1:**
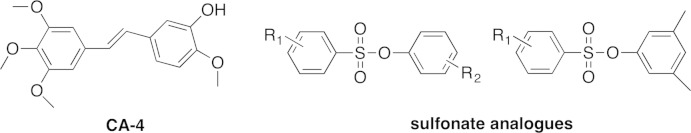
The structures of CA-4 and sulfonate analogues, where *R*_1_ and *R*_2_ are substituents on the sulfonyl and phen­oxy benzene rings, respectively.

Research in the field for the synthesis of new anti­microtubule compounds has been geared towards compounds with improved biopharmaceutical properties (Fortin *et al.*, 2011 ▸). To this end, Fortin and co-workers have designed and synthesized various sulfonate derivatives, which have been tested as new tubulin inhibitors mimicking Combretastatin A-4 (Fig. 1 ▸).

A series of sulfonate derivatives have shown promise as anti­cancer drugs, with some having lower toxicity than CA-4 (Fortin *et al.*, 2011 ▸). We embarked on the synthesis of sulfonate derivatives with the long-term goal of investigating the effect of the benzene-ring substituents on the cytotoxicity of the sulfonate derivatives. To the best of our knowledge, despite the simplicity of the sulfonate derivatives, there has been no relevant previous crystallographic studies. Therefore, we report here the synthesis and crystal structure of 3,5-di­methyl­phenyl 2-nitro­benzene­sulfonate.

## Structural commentary   

**Figure 2:**
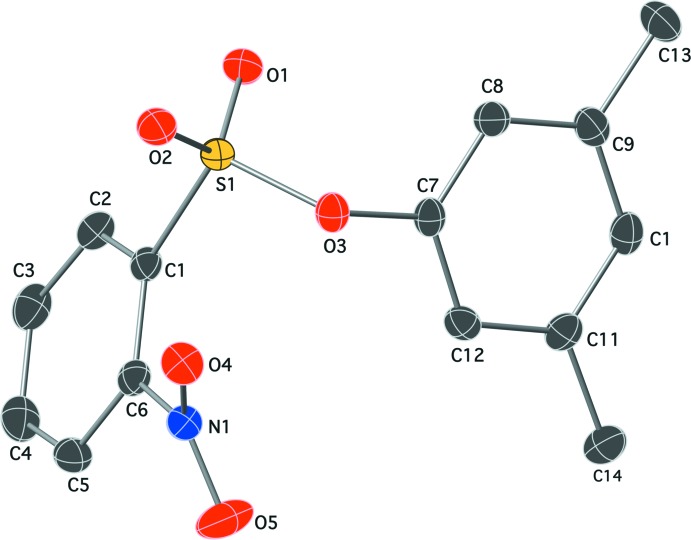
The mol­ecular structure of the title compound, with displacement ellipsoids shown at the 50% probability level. All H atoms have been omitted for clarity. Color codes: black C, blue N, red O and yellow S.

In the title mol­ecule (Fig. 2 ▸), the O1=S1=O2 and C1—S1—O3 bond angles of 119.41 (7) and 104.16 (6)° are typical for phenyl benzene­sulfonates with a *gauche* conformation around the ester S—O bond. The torsion angle C1—S1—O3—C7 around the ester bond is −84.68 (11)°. Owing to steric hindrance between the *ortho* substituents of the benzene ring, the nitro group is twisted relative to the benzene best plane by 39.91 (2)°, so that the shortest contact of 2.7941 (16) Å between the O atoms of these groups is close to the sum of the van der Waals radii.

## Supra­molecular features   

**Figure 3:**
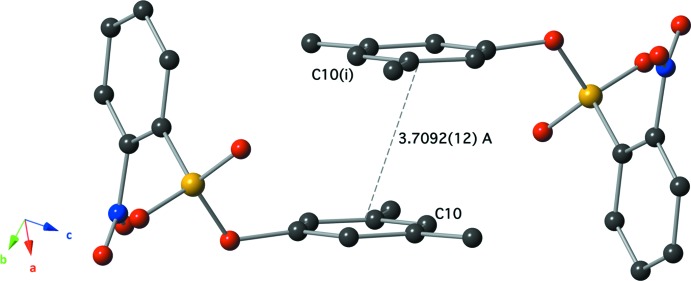
The centrosymmetric dimers formed by inter­molecular offset π–π stacking inter­actions. [Symmetry code: (i) −*x* + 1, −*y* + 2, −*z*.]

**Figure 4:**
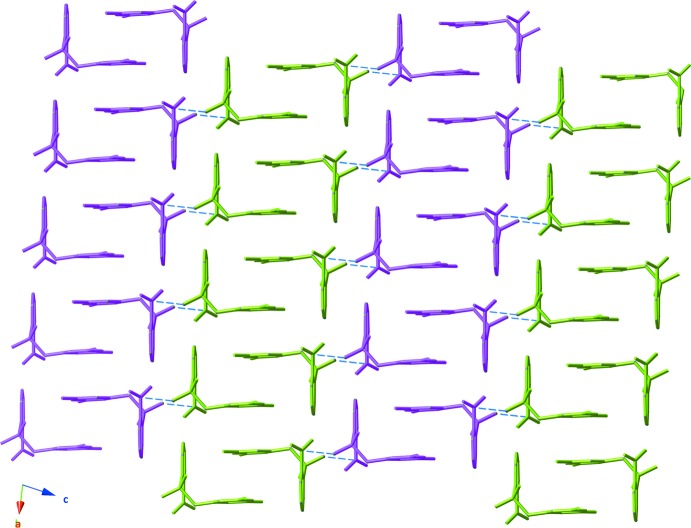
The packing of mol­ecules in the crystal, viewed down the [110] direction. Columns of dimers formed *via* stacking inter­actions are colored green and pink in an alternating fashion, and potential N⋯O=S inter­actions are denoted with blue dashed lines.

The mol­ecules of the title compound form centrosymmetric dimers *via* inter­molecular π–π stacking inter­actions between the relatively electron-rich C7–C12 benzene rings (Fig. 3 ▸), with a plane-to-plane distance of 3.4147 (15) Å. The aromatic rings are stacked with an offset, and the distance between the centroids of these rings is 3.709 (12) Å. Another centrosymmetric dimer is formed by an S=O⋯N inter­action, with an N1⋯O2 inter­atomic distance of 2.9840 (18) Å. O⋯N(nitro) inter­actions between nitro groups have been discussed in the literature (Daszkiewicz, 2013 ▸; Caracelli *et al.*, 2014 ▸) and we report here the case of sulfonyl and nitro group inter­actions. Both types of dimers are assembled into a column-type structure extending along [011] (Fig. 4 ▸).

There are no classical hydrogen bonds in the crystal structure; however, nonclassical C—H⋯O inter­actions between aromatic-ring H atoms and sulfonyl and nitro group O atoms organize the [011] columns into (111) layers. The geometry of these inter­actions is given in Table 1 ▸.

## Database survey   

The Cambridge Structural Database (CSD, Version 5.36 with two updates; Groom & Allen, 2014 ▸) contains three structures with an *o*-nitro­aryl­sulfonyl moiety bonded to an aromatic ring through an ester linkage. These are CSD refcodes FEMQUK (Ichikawa *et al.*, 2004 ▸), MIBZUT (Pelly *et al.*, 2007 ▸), and FEMRIZ (Ichikawa *et al.*, 2004 ▸). Like in the title compound, the aromatic substituents of the SO_3_ group are oriented *gauche* around the ester S—O bond and the absolute value of the C—S—O—C torsion angle is in the range 85.9 (3)–103.43 (13)°. In each of these structures there are either intra- or inter­molecular S=O⋯N inter­actions between the sulfonate and *o*-nitro groups.

## Synthesis and crystallization   

3,5-Di­methyl­phenol (2.44 g, 20 mmol) was dissolved in chilled di­chloro­methane (25 ml). This was followed by the addition of pyridine (3.2 ml, 40 mmol). The resulting solution was cooled in an ice bath under an N_2_ atmosphere, followed by the addition of 2-nitro­benzene­sulfonyl chloride (4.43 g, 20 mmol) portion-wise. The mixture was stirred at 273 K for 30 mins and then at room temperature for 24 h. The product precipitated from the reaction mixture after sitting at 277 K for two weeks. The product was redissolved in di­chloro­methane, and the solvent was allowed to evaporate slowly to give large block-shaped crystals that were suitable for analysis by X-ray diffraction (m.p. 374–378 K).

## Refinement   

Crystal data, data collection and structure refinement details are summarized in Table 2 ▸. H atoms were placed in calculated positions and constrained to ride on their parent atoms, with *U*_iso_(H) = 1.2*U*_eq_(C) for CH groups and 1.5*U*_eq_(C) for methyl groups.

## Supplementary Material

Crystal structure: contains datablock(s) I. DOI: 10.1107/S2056989015015078/gk2639sup1.cif

Structure factors: contains datablock(s) I. DOI: 10.1107/S2056989015015078/gk2639Isup2.hkl

Supporting information file. DOI: 10.1107/S2056989015015078/gk2639Isup3.cml

CCDC reference: 1418463

Additional supporting information:  crystallographic information; 3D view; checkCIF report

## Figures and Tables

**Table 1:** Hydrogen-bond geometry (, )

*D*H*A*	*D*H	H*A*	*D* *A*	*D*H*A*
C5H5O1^i^	0.95	2.56	3.4544(19)	156
C8H8O5^ii^	0.95	2.56	3.468(2)	160

Symmetry codes: (i) 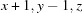; (ii) 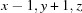.

**Table 2:** Experimental details

Crystal data	
Chemical formula	C_14_H_13_NO_5_S
*M*_r_	307.31
Crystal system, space group	Triclinic, *P* 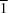
Temperature (K)	173
*a*, *b*, *c* ()	7.9958(4), 7.9991(5), 12.0238(3)
, , ()	83.908(3), 76.286(3), 63.411(4)
*V* (^3^)	668.10(6)
*Z*	2
Radiation type	Cu *K*
(mm^1^)	2.37
Crystal size (mm)	0.38 0.34 0.21

Data collection	
Diffractometer	Bruker APEXII CCD
Absorption correction	Multi-scan (*SADABS*; Bruker, 2013 ▸)
*T*_min_, *T*_max_	0.630, 0.754
No. of measured, independent and observed [*I* > 2(*I*)] reflections	10317, 2519, 2461
*R*_int_	0.022
(sin /)_max_ (^1^)	0.617

Refinement	
*R*[*F* ^2^ > 2(*F* ^2^)], *wR*(*F* ^2^), *S*	0.035, 0.092, 1.05
No. of reflections	2519
No. of parameters	192
H-atom treatment	H-atom parameters constrained
_max_, _min_ (e ^3^)	0.33, 0.50

Computer programs: *APEX2* and *SAINT* (Bruker, 2013 ▸), *SHELXS97* (Sheldrick, 2008 ▸), *SHELXL2014* (Sheldrick, 2015 ▸), *CrystalMaker* (Palmer, 2007 ▸) and *OLEX2* (Dolomanov *et al.*, 2009 ▸; Bourhis *et al.*, 2015 ▸).

## supplementary crystallographic information

### Crystal data

**Table:**

C_14_H_13_NO_5_S	*Z* = 2
*M**_r_* = 307.31	*F*(000) = 320
Triclinic, *P*1	*D*_x_ = 1.528 Mg m^−^^3^
*a* = 7.9958 (4) Å	Cu *K*α radiation, λ = 1.54178 Å
*b* = 7.9991 (5) Å	Cell parameters from 8863 reflections
*c* = 12.0238 (3) Å	θ = 3.8–72.3°
α = 83.908 (3)°	µ = 2.37 mm^−^^1^
β = 76.286 (3)°	*T* = 173 K
γ = 63.411 (4)°	Block, colourless
*V* = 668.10 (6) Å^3^	0.38 × 0.34 × 0.21 mm

### Data collection

**Table:**

Bruker APEXII CCD diffractometer	2519 independent reflections
Radiation source: sealed tube	2461 reflections with *I* > 2σ(*I*)
Graphite monochromator	*R*_int_ = 0.022
Detector resolution: 8 pixels mm^-1^	θ_max_ = 72.1°, θ_min_ = 3.8°
φ and ω scans	*h* = −9→9
Absorption correction: multi-scan (*SADABS*; Bruker, 2013)	*k* = −9→9
*T*_min_ = 0.630, *T*_max_ = 0.754	*l* = −14→14
10317 measured reflections	

### Refinement

**Table:**

Refinement on *F*^2^	Primary atom site location: structure-invariant direct methods
Least-squares matrix: full	Hydrogen site location: inferred from neighbouring sites
*R*[*F*^2^ > 2σ(*F*^2^)] = 0.035	H-atom parameters constrained
*wR*(*F*^2^) = 0.092	*w* = 1/[σ^2^(*F*_o_^2^) + (0.0559*P*)^2^ + 0.3536*P*] where *P* = (*F*_o_^2^ + 2*F*_c_^2^)/3
*S* = 1.05	(Δ/σ)_max_ < 0.001
2519 reflections	Δρ_max_ = 0.33 e Å^−^^3^
192 parameters	Δρ_min_ = −0.50 e Å^−^^3^
0 restraints	

### Special details

**Table:**

Geometry. All e.s.d.'s (except the e.s.d. in the dihedral angle between two l.s. planes) are estimated using the full covariance matrix. The cell e.s.d.'s are taken into account individually in the estimation of e.s.d.'s in distances, angles and torsion angles; correlations between e.s.d.'s in cell parameters are only used when they are defined by crystal symmetry. An approximate (isotropic) treatment of cell e.s.d.'s is used for estimating e.s.d.'s involving l.s. planes.

### Fractional atomic coordinates and isotropic or equivalent isotropic displacement parameters (Å^2^)

**Table:**

	*x*	*y*	*z*	*U*_iso_*/*U*_eq_	
S1	0.32156 (5)	0.85859 (5)	0.36621 (3)	0.01694 (13)	
O1	0.27175 (16)	1.05073 (15)	0.33973 (10)	0.0239 (3)	
O2	0.22079 (15)	0.81004 (16)	0.46826 (9)	0.0240 (3)	
O3	0.29830 (15)	0.76150 (15)	0.26599 (9)	0.0193 (2)	
O4	0.46725 (16)	0.43665 (16)	0.39045 (10)	0.0240 (3)	
O5	0.73130 (19)	0.28883 (18)	0.26840 (12)	0.0375 (3)	
N1	0.62367 (18)	0.41785 (18)	0.33520 (11)	0.0201 (3)	
C1	0.5719 (2)	0.7475 (2)	0.36177 (12)	0.0171 (3)	
C2	0.6508 (2)	0.8662 (2)	0.37453 (13)	0.0216 (3)	
H2	0.5728	0.9973	0.3801	0.026*	
C3	0.8425 (2)	0.7951 (3)	0.37920 (14)	0.0258 (4)	
H3	0.8939	0.8771	0.3902	0.031*	
C4	0.9587 (2)	0.6047 (3)	0.36792 (14)	0.0265 (4)	
H4	1.0898	0.5564	0.3711	0.032*	
C5	0.8841 (2)	0.4845 (2)	0.35195 (13)	0.0227 (3)	
H5	0.9643	0.3542	0.3426	0.027*	
C6	0.6920 (2)	0.5556 (2)	0.34977 (12)	0.0182 (3)	
C7	0.3244 (2)	0.8273 (2)	0.15112 (12)	0.0175 (3)	
C8	0.1844 (2)	0.9933 (2)	0.12119 (13)	0.0200 (3)	
H8	0.0770	1.0683	0.1773	0.024*	
C9	0.2044 (2)	1.0482 (2)	0.00677 (14)	0.0214 (3)	
C10	0.3619 (2)	0.9314 (2)	−0.07306 (13)	0.0217 (3)	
H10	0.3744	0.9677	−0.1513	0.026*	
C11	0.5014 (2)	0.7635 (2)	−0.04207 (13)	0.0198 (3)	
C12	0.4822 (2)	0.7111 (2)	0.07297 (13)	0.0186 (3)	
H12	0.5755	0.5979	0.0971	0.022*	
C13	0.0582 (3)	1.2305 (2)	−0.02968 (16)	0.0294 (4)	
H13A	−0.0609	1.2720	0.0288	0.044*	
H13B	0.0333	1.2131	−0.1026	0.044*	
H13C	0.1069	1.3249	−0.0390	0.044*	
C14	0.6690 (2)	0.6430 (2)	−0.13189 (14)	0.0249 (3)	
H14A	0.6251	0.6435	−0.2015	0.037*	
H14B	0.7263	0.5147	−0.1032	0.037*	
H14C	0.7647	0.6923	−0.1494	0.037*	

### Atomic displacement parameters (Å^2^)

**Table:**

	*U*^11^	*U*^22^	*U*^33^	*U*^12^	*U*^13^	*U*^23^
S1	0.01488 (19)	0.0172 (2)	0.0159 (2)	−0.00464 (15)	−0.00261 (13)	−0.00112 (14)
O1	0.0254 (6)	0.0177 (6)	0.0258 (6)	−0.0055 (4)	−0.0081 (5)	−0.0009 (5)
O2	0.0186 (5)	0.0292 (6)	0.0188 (5)	−0.0075 (5)	−0.0002 (4)	0.0000 (5)
O3	0.0225 (5)	0.0206 (5)	0.0174 (5)	−0.0113 (4)	−0.0061 (4)	0.0026 (4)
O4	0.0227 (6)	0.0257 (6)	0.0254 (6)	−0.0129 (5)	−0.0043 (5)	0.0023 (5)
O5	0.0340 (7)	0.0311 (7)	0.0430 (8)	−0.0111 (6)	0.0021 (6)	−0.0198 (6)
N1	0.0215 (6)	0.0181 (6)	0.0186 (6)	−0.0063 (5)	−0.0052 (5)	0.0000 (5)
C1	0.0157 (7)	0.0211 (7)	0.0126 (6)	−0.0072 (6)	−0.0014 (5)	−0.0007 (6)
C2	0.0236 (7)	0.0231 (8)	0.0179 (7)	−0.0108 (6)	−0.0017 (6)	−0.0027 (6)
C3	0.0258 (8)	0.0362 (9)	0.0219 (8)	−0.0194 (7)	−0.0024 (6)	−0.0044 (7)
C4	0.0170 (7)	0.0397 (10)	0.0227 (8)	−0.0124 (7)	−0.0034 (6)	−0.0006 (7)
C5	0.0183 (7)	0.0240 (8)	0.0195 (7)	−0.0046 (6)	−0.0021 (6)	−0.0001 (6)
C6	0.0187 (7)	0.0212 (7)	0.0137 (7)	−0.0087 (6)	−0.0017 (5)	−0.0006 (6)
C7	0.0210 (7)	0.0189 (7)	0.0169 (7)	−0.0116 (6)	−0.0064 (6)	0.0016 (6)
C8	0.0191 (7)	0.0189 (7)	0.0226 (8)	−0.0074 (6)	−0.0060 (6)	−0.0029 (6)
C9	0.0244 (7)	0.0177 (7)	0.0256 (8)	−0.0098 (6)	−0.0114 (6)	0.0021 (6)
C10	0.0304 (8)	0.0213 (8)	0.0181 (7)	−0.0145 (7)	−0.0086 (6)	0.0030 (6)
C11	0.0238 (7)	0.0188 (7)	0.0208 (7)	−0.0127 (6)	−0.0038 (6)	−0.0023 (6)
C12	0.0196 (7)	0.0146 (7)	0.0233 (8)	−0.0077 (6)	−0.0073 (6)	0.0010 (6)
C13	0.0324 (9)	0.0217 (8)	0.0327 (9)	−0.0074 (7)	−0.0158 (7)	0.0046 (7)
C14	0.0288 (8)	0.0225 (8)	0.0230 (8)	−0.0121 (7)	−0.0017 (6)	−0.0035 (6)

### Geometric parameters (Å, º)

**Table:**

S1—O1	1.4249 (12)	C7—C8	1.380 (2)
S1—O2	1.4198 (11)	C7—C12	1.383 (2)
S1—O3	1.5887 (11)	C8—H8	0.9500
S1—C1	1.7797 (15)	C8—C9	1.393 (2)
O3—C7	1.4268 (18)	C9—C10	1.394 (2)
O4—N1	1.2195 (17)	C9—C13	1.505 (2)
O5—N1	1.2263 (18)	C10—H10	0.9500
N1—C6	1.473 (2)	C10—C11	1.392 (2)
C1—C2	1.391 (2)	C11—C12	1.395 (2)
C1—C6	1.400 (2)	C11—C14	1.507 (2)
C2—H2	0.9500	C12—H12	0.9500
C2—C3	1.389 (2)	C13—H13A	0.9800
C3—H3	0.9500	C13—H13B	0.9800
C3—C4	1.385 (3)	C13—H13C	0.9800
C4—H4	0.9500	C14—H14A	0.9800
C4—C5	1.387 (2)	C14—H14B	0.9800
C5—H5	0.9500	C14—H14C	0.9800
C5—C6	1.385 (2)		
			
O1—S1—O3	109.83 (6)	C8—C7—C12	123.31 (14)
O1—S1—C1	106.85 (7)	C12—C7—O3	117.36 (13)
O2—S1—O1	119.41 (7)	C7—C8—H8	120.8
O2—S1—O3	105.16 (6)	C7—C8—C9	118.42 (14)
O2—S1—C1	110.43 (7)	C9—C8—H8	120.8
O3—S1—C1	104.16 (6)	C8—C9—C10	118.82 (14)
C7—O3—S1	120.43 (9)	C8—C9—C13	120.43 (15)
O4—N1—O5	124.23 (14)	C10—C9—C13	120.75 (15)
O4—N1—C6	118.87 (12)	C9—C10—H10	118.9
O5—N1—C6	116.89 (13)	C11—C10—C9	122.27 (15)
C2—C1—S1	115.24 (12)	C11—C10—H10	118.9
C2—C1—C6	118.36 (14)	C10—C11—C12	118.57 (14)
C6—C1—S1	126.38 (12)	C10—C11—C14	120.02 (14)
C1—C2—H2	119.7	C12—C11—C14	121.41 (14)
C3—C2—C1	120.67 (15)	C7—C12—C11	118.60 (14)
C3—C2—H2	119.7	C7—C12—H12	120.7
C2—C3—H3	119.9	C11—C12—H12	120.7
C4—C3—C2	120.11 (15)	C9—C13—H13A	109.5
C4—C3—H3	119.9	C9—C13—H13B	109.5
C3—C4—H4	119.9	C9—C13—H13C	109.5
C3—C4—C5	120.10 (15)	H13A—C13—H13B	109.5
C5—C4—H4	119.9	H13A—C13—H13C	109.5
C4—C5—H5	120.2	H13B—C13—H13C	109.5
C6—C5—C4	119.59 (15)	C11—C14—H14A	109.5
C6—C5—H5	120.2	C11—C14—H14B	109.5
C1—C6—N1	122.82 (13)	C11—C14—H14C	109.5
C5—C6—N1	116.05 (14)	H14A—C14—H14B	109.5
C5—C6—C1	121.13 (14)	H14A—C14—H14C	109.5
C8—C7—O3	119.06 (13)	H14B—C14—H14C	109.5
			
S1—O3—C7—C8	−74.03 (15)	C1—S1—O3—C7	−84.68 (11)
S1—O3—C7—C12	111.80 (13)	C1—C2—C3—C4	1.8 (2)
S1—C1—C2—C3	176.26 (12)	C2—C1—C6—N1	−179.70 (14)
S1—C1—C6—N1	2.0 (2)	C2—C1—C6—C5	0.8 (2)
S1—C1—C6—C5	−177.44 (12)	C2—C3—C4—C5	0.0 (2)
O1—S1—O3—C7	29.44 (12)	C3—C4—C5—C6	−1.3 (2)
O1—S1—C1—C2	21.24 (13)	C4—C5—C6—N1	−178.57 (13)
O1—S1—C1—C6	−160.46 (13)	C4—C5—C6—C1	0.9 (2)
O2—S1—O3—C7	159.13 (10)	C6—C1—C2—C3	−2.2 (2)
O2—S1—C1—C2	−110.09 (12)	C7—C8—C9—C10	1.6 (2)
O2—S1—C1—C6	68.21 (15)	C7—C8—C9—C13	−178.43 (14)
O3—S1—C1—C2	137.46 (11)	C8—C7—C12—C11	0.0 (2)
O3—S1—C1—C6	−44.23 (14)	C8—C9—C10—C11	−1.1 (2)
O3—C7—C8—C9	−174.87 (12)	C9—C10—C11—C12	0.0 (2)
O3—C7—C12—C11	173.85 (12)	C9—C10—C11—C14	−179.82 (14)
O4—N1—C6—C1	−39.7 (2)	C10—C11—C12—C7	0.6 (2)
O4—N1—C6—C5	139.82 (14)	C12—C7—C8—C9	−1.1 (2)
O5—N1—C6—C1	141.33 (16)	C13—C9—C10—C11	178.94 (14)
O5—N1—C6—C5	−39.2 (2)	C14—C11—C12—C7	−179.61 (13)

### Hydrogen-bond geometry (Å, º)

**Table:**

*D*—H···*A*	*D*—H	H···*A*	*D*···*A*	*D*—H···*A*
C5—H5···O1^i^	0.95	2.56	3.4544 (19)	156
C8—H8···O5^ii^	0.95	2.56	3.468 (2)	160

Symmetry codes: (i) *x*+1, *y*−1, *z*; (ii) *x*−1, *y*+1, *z*.
